# Ribosomal RNA gene repeats associate with the nuclear pore complex for maintenance after DNA damage

**DOI:** 10.1371/journal.pgen.1008103

**Published:** 2019-04-18

**Authors:** Chihiro Horigome, Eri Unozawa, Takamasa Ooki, Takehiko Kobayashi

**Affiliations:** 1 Laboratory of Genome Regeneration, Institute for Quantitative Biosciences (IQB), Bunkyo-ku, Japan; 2 Institute of Molecular and Cellular Biosciences, The University of Tokyo, Bunkyo-ku, Japan; 3 National Institute of Genetics, Shizuoka, Japan; 4 Sokendai, Yata, Mishima, Shizuoka, Japan; 5 Department of Biological Sciences, Graduate School of Science, The University of Tokyo, Hongo, Bunkyo-ku, Japan; 6 Collaborative Research Institute for Innovative Microbiology, The University of Tokyo, Bunkyo-ku, Japan; Columbia University, UNITED STATES

## Abstract

The ribosomal RNA genes (rDNA) comprise a highly repetitive gene cluster. The copy number of genes at this locus can readily change and is therefore one of the most unstable regions of the genome. DNA damage in rDNA occurs after binding of the replication fork blocking protein Fob1 in S phase, which triggers unequal sister chromatid recombination. However, the precise mechanisms by which such DNA double-strand breaks (DSBs) are repaired is not well understood. Here, we demonstrate that the conserved protein kinase Tel1 maintains rDNA stability after replication fork arrest. We show that rDNA associates with nuclear pores, which is dependent on DNA damage checkpoint kinases Mec1/Tel1 and replisome component Tof1. These findings suggest that rDNA-nuclear pore association is due to a replication fork block and subsequent DSB. Indeed, quantitative microscopy revealed that rDNA is relocated to the nuclear periphery upon induction of a DSB. Finally, rDNA stability was reduced in strains where this association with the nuclear envelope was prevented, which suggests its importance for avoiding improper recombination repair that could induce repeat instability.

## Author summary

Ribosomal RNA genes (rDNA) comprise an unstable region of the genome due to their highly repetitive structure and elevated levels of transcription. Collision between transcription and replication machineries of rDNA, which may lead to DNA damage in the form of a double-stranded break, is avoided by the replication fork barrier. When such a break is repaired by homologous recombination with a repeat on the sister chromatid, the abundance of homologous sequences may lead to a change in copy number. In most organisms, however, only small variations in copy number are observed, indicating that the rDNA is stably maintained. Our results suggest that some parts of rDNA become localized to the nuclear pore complex in a DNA double-strand break-dependent manner. This localization requires the protein kinase Tel1, which is involved in the DNA damage response pathway, and factors that recruit condensin, which facilitates condensation and segregation of rDNA during mitosis. We found that the rDNA becomes unstable when association with the nuclear envelope was prevented. Thus, the localization represents a unique strategy for maintaining repeat integrity after DNA damage.

## Introduction

DNA damage can lead to deletion, translocation and amplification of DNA in the genome, which may result in cell death, cancer and cellular senescence [1]. The most hazardous forms of genomic damage is the DNA double-strand break (DSB) that can occur randomly in the chromosome during replication, mainly in the S phase of the cell cycle, when the replication fork is arrested by DNA damage, torsional stress, modified nucleotides, or colliding transcription complexes. Stalled replication forks are thought to be targets of endonucleases that induce a DSB [2]. Downstream events of a DSB, such as DNA damage checkpoint control and DSB repair, have been analyzed [3]. Nonetheless, the mechanism of DSB repair in repetitive sequences without rearrangement is not well understood. Insights into the cellular mechanisms that prevent these rearrangements while allowing the broken genome to be repaired will contribute to the development of novel cancer treatments and broaden our understanding of the aging process.

**Fig 1:**
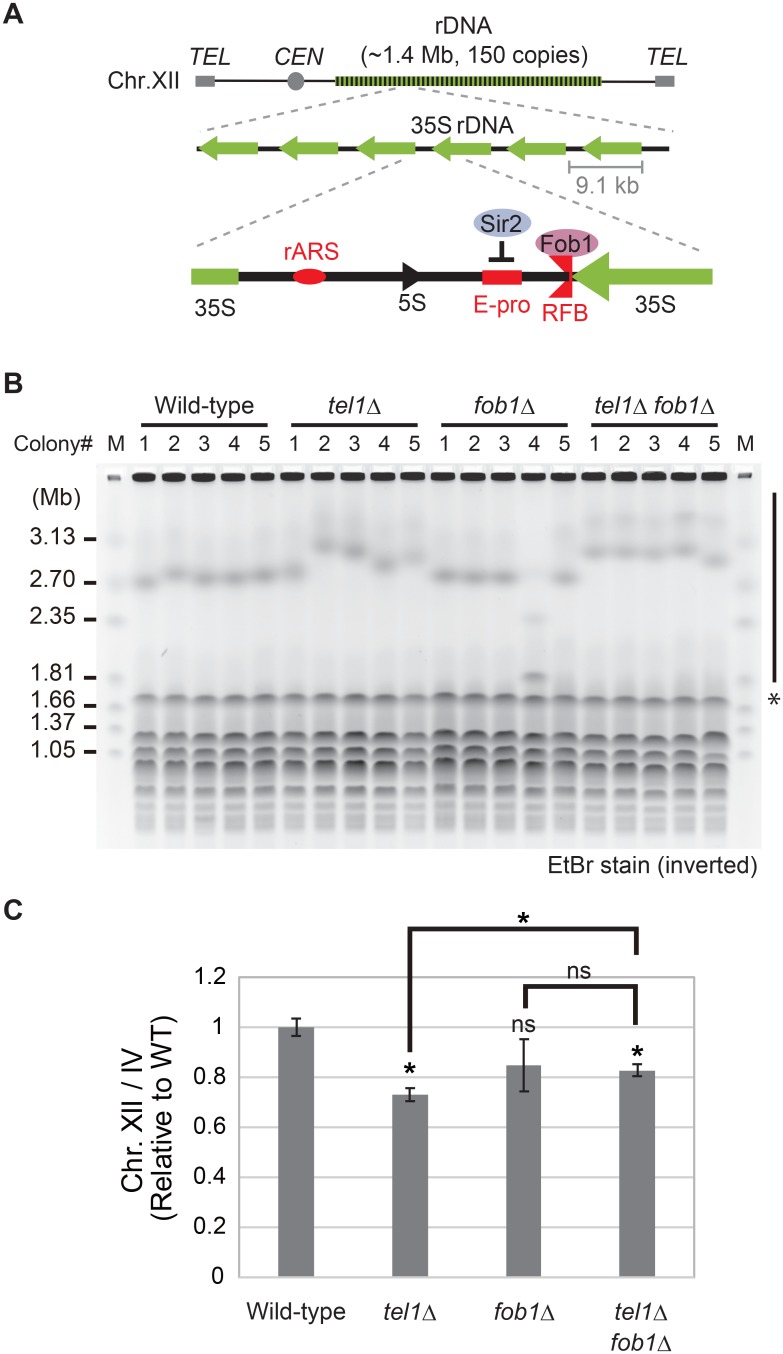
Analysis of rDNA stability in the *tel1*Δ mutant. (A) The structure of rDNA in the yeast *Saccharomyces cerevisiae*. In budding yeast, there are about 150 tandemly repeated copies of the rDNA on chromosome XII. An rDNA repeating unit consists of two rRNA genes (5S and 35S). In the intergenic spacer regions, there is a replication origin (rARS), a replication fork barrier (RFB) site and a non-coding promoter (E-pro). Fob1, a replication fork blocking protein, binds to the RFB site and Sir2, a histone deacetylase, represses E-pro transcription. *TEL*; telomere, *CEN*; centromere. (B) Pulsed field gel electrophoresis for assessing rDNA stability in the *tel1*Δ and *tel1*Δ *fob1*Δ mutants, and the gels were stained with ethidium bromide (EtBr) for visualization. A side bar and an asterisks mark the position of chromosome XII and IV, respectively. M is the size marker (*H*. *wingei* chromosomes). (C) Quantitation of rDNA instability shown in B. Signal intensities of the Chr. XII bands in a fixed square area that fits the size of Chr. IV were measured and normalized to that of Chr. IV (see Materials and methods). The values are relative to that in the wild-type strain. Error bars show the standard error (SEM) of five independent colonies. The significance levels (* *p* < 0.05) are from the unpaired two-tailed *t*-tests. ns, not significant. *P*-values are shown in S2B Table.

Here, we focus on the ribosomal RNA gene repeat (rDNA) to investigate the mechanism by which genome rearrangement is prevented after a DSB at a site with a stalled replication fork. In eukaryotic cells the rDNA forms a huge, conserved, tandem repeating structure (> 100 copies) on the chromosome. Transcription at this locus generates ribosomal RNA (rRNA) that, together with the ribosomal proteins, is assembled into ribosomes. A large number of ribosomes are needed to sustain cell-growth. Indeed, rRNA comprises approximately 80% of the total RNA in a cell [4] and, in the case of budding yeast *Saccharomyces cerevisiae*, ~ 150 rDNA copies are present on chromosome XII. Each repeating unit contains 35S and 5S rRNA genes, which are transcribed by RNA polymerases I and III, respectively (Fig 1A). The transcript of the 35S rRNA gene is subsequently processed into mature 5.8S, 18S and 25S rRNA.

The stability of rDNA is affected by recombination among the repeats, which can be easily detected by pulsed field gel electrophoresis [5]. For the upkeep of repeat number, cells can use a gene amplification mechanism that helps to maintain copy number by recombination [6]. In this system, replication is arrested at the replication fork barrier (RFB) site, located near the 3’ termination site of the 35S ribosomal RNA gene (Fig 1A and S1 Fig). A complex formed by the binding of Fob1 to the RFB site inhibits replication against the direction of rDNA transcription [7]. A DSB is subsequently induced at the RFB site (~6% of arrested forks at the RFB site result in a DSB) and repaired by recombination with the sister-chromatid [5, 8, 9]. When the broken end recombines unequally with a homologous site on the sister chromatid and replication restarts, some copies are replicated twice resulting in an increased copy number (S1B-1 Fig). Thus, cells can use the rearrangement for copy number maintenance.

This mechanism is regulated by the interplay between Sir2, a histone deacetylase, and transcription from the nearby bidirectional promoter E-pro (S1 Fig). In a cell with a wild-type rDNA copy number (~150), E-pro transcription is repressed by Sir2, but this repression does not occur in cells with a low rDNA copy number [10]. Non-coding transcription from E-pro, which prevents sister-chromatid cohesion, stimulates unequal sister-chromatid recombination [8]. When the copy number reaches the wild-type level, amplification stops. Alternatively, a DSB in the rDNA of a strain with a normal copy number can be repaired by a mechanism that does not involve homologous recombination, which reduces the risk of rearrangement (and thus copy number instability). In this mechanism, as we have shown recently, a replisome component Ctf4 protects arrested forks from breakage and end resection. Although this pathway needs to be elucidated in more detail, it appears that DSB repair at arrested forks is regulated differently from replication-independent DSBs [9].

By using the unstable nature of rDNA as a measure, we screened a yeast library of ~4,800 deletion mutants of non-essential genes and identified ~700 ribosomal RNA gene unstable mutants (RiUMs) [11, 12] (http://lafula-com.info/kobayashiken/geldata/index.php). Among the RiUMs there was a deletion in *TEL1*, which is an orthologue of the human ataxia-telangiectasia mutated (ATM) gene that responds to DNA damage and functions in telomere maintenance, damage checkpoint control and DSB repair [13]. Ataxia-telangiectasia or Louis–Bar syndrome is a rare, neurodegenerative, autosomal recessive disease that causes severe disability. In budding yeast, Tel1 regulates telomere length through phosphorylation of proteins involved in DSB repair and promotes elongation of telomere repeats [14]. Although Tel1 functions redundantly with the ATR orthologue Mec1 as S phase checkpoint kinases (reviewed in [15]), the function of these proteins in rDNA maintenance has not been determined.

Certain types of DNA repair appear to arise through recruitment of damage to specific subnuclear sites (reviewed in [16]). *TEL1* is involved in the relocation of DNA to the nuclear pores after inducing DSBs by means of endonuclease HO during the G1 and S/G2-phases of the cell cycle [17]. This irreparably damaged DNA also binds to the essential Sad1/UNC-84 (SUN) domain protein Mps3 in the inner nuclear membrane, but only when DSBs are induced during the S/G2-phase [18–20].

The rDNA instability in *tel1*Δ observed in our screen prompted us to investigate whether naturally occurring DSBs formed after replication arrest cause rDNA to translocate to the nuclear envelope. Using chromatin immunoprecipitation (ChIP) assays, we detected binding of rDNA to the nuclear pores, which required Tel1 and Mec1, indicating this localization is DNA-damage dependent. In addition, Tof1, a component of the replisome, which is necessary for fork arrest at the RFB, together with condensin recruiting factors were also found to be required for localization of rDNA to the nuclear pores. Defective association to nuclear pores reduced rDNA stability, suggesting that this association helps to maintain repeat stability.

## Results

### Tel1 maintains rDNA stability after replication fork arrest

Recently, we screened a yeast deletion library for factors involved in the maintenance of rDNA stability and identified ~700 ribosomal RNA unstable mutants (RiUM) [11, 12]. Among these, there were genes related to DNA repair for which the molecular mechanism with respect to rDNA was not known. In this category, we focused on a protein kinase Tel1 that regulates telomere length through phosphorylation of proteins mediating DSB repair and that enhance elongation of telomere repeats [14]. We first introduced the *tel1* deletion to our laboratory strain to confirm the generality of the phenotype. We performed PFGE assays three times and one of the trials was followed by Southern blotting with an rDNA probe (Fig 1B, S2A and S2B Fig). Although the effect was relatively modest as that of the library strain, quantitative analysis revealed that the bands of rDNA-containing chromosome XII were broader in the *tel1*Δ compared to wild-type (Fig 1C. See S2B Table and Materials and methods for about the quantification). Such variable copy numbers are a hallmark of unstable rDNA [5]. In this assay, the bands of chromosome XII in *fob1*Δ were not shaper compared to wild-type. The similar observation was made in a previous study illustrating the inherent difficulty of the detection of a more stable band than that of the wild-type strain [12].

To test whether rDNA instability in the *tel1*Δ is related to replication fork barrier activity that induces a DSB, we made a double mutant, *tel1*Δ *fob1*Δ. In the double mutant, the bands of chromosome XII became as sharp as that of the *fob1*Δ (Fig 1B and 1C), indicating that rDNA instability in the *tel1*Δ is caused downstream of Fob1. Thus, Tel1 functions after replication fork arrest mediated by Fob1 and before involvement in rDNA maintenance.

### Tel1 does not affect RFB activity and DSB frequency

**Fig 2:**
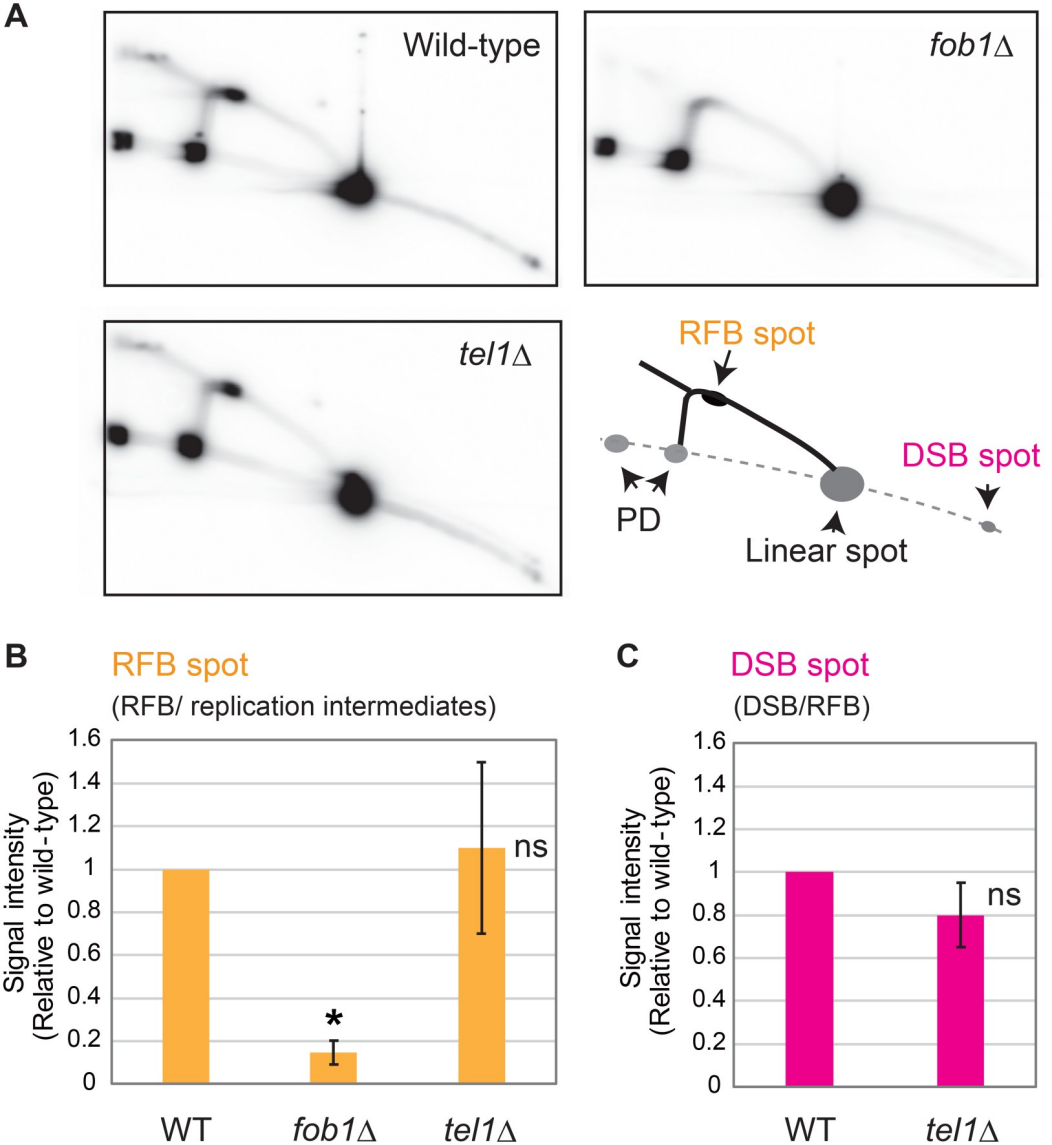
Two-dimensional gel electrophoresis (2D analysis) for detecting RFB and DSB intermediates. (A) 2D analysis in the *tel1*Δ and *fob1*Δ. The rDNA was detected with an rDNA specific probe. A schematic explanation is shown on the bottom right. The solid black line (Y-arc and Double Y) and spot (RFB-spot) are replication intermediates. PD is the signal of partial digestion by *Bgl*II. DSB-spot is the signal of broken fragments at the RFB site. (B and C) Quantitation of the signal intensity of RFB- and DSB-spots (B and C, respectively). The RFB- and DSB-spot signals were normalized to those of total replication intermediates and RFB-spot signals, respectively. The values (average of three experiments) are relative to the wild-type and standard errors (SEM) are shown. The significance levels (* *p* < 0.05) are from the unpaired two-tailed *t*-tests. ns, not significant.

We reasoned *tel1*Δ might have an effect on replication fork blocking activity and therefore DSB frequency at the RFB site. Thus, we examined this possibility by two dimensional gel electrophoresis (2D gel assay) in which the amount of replication fork arrest can be determined from the signal intensity of the “RFB-spot” corresponding to the number of Y-shaped replication intermediates accumulating at the RFB site [21, 22]. In the *tel1*Δ, the “Y-arc, Double-Y and RFB-spot” signals, corresponding to replication intermediates, was slightly weaker than that in the wild-type cells, probably because of the reduced number of S-phase cells in the mutant (Fig 2A). To compare these strains, RFB-spot intensity was normalized to the replication intermediates. No significant difference in stalling of the replication forks was observed (RFB-spot, Fig 2A and 2B and S2B Table). The 2D gel-assay also gave insight into the frequency with which a DSB is formed after replication-fork arrest by means of the “DSB-spot” i.e. a signal that corresponds to broken fragments at the RFB site. The signal of the spot (~2.3 kb) disappeared in the *fob1*Δ because there was no arrest of the replication fork [8, 23]. Relative to the RFB spot, the intensity of the DSB spot was not affected in the *tel1*Δ (Fig 2A and 2C and S2B Table). Thus, the increased levels of replication fork blocking activity and resulting increased DSBs are unlikely to be the cause of rDNA instability in the *tel1*Δ.

### rDNA is associated with nuclear pores in a Mec1, Tel1 and Tof1 dependent manner

**Fig 3:**
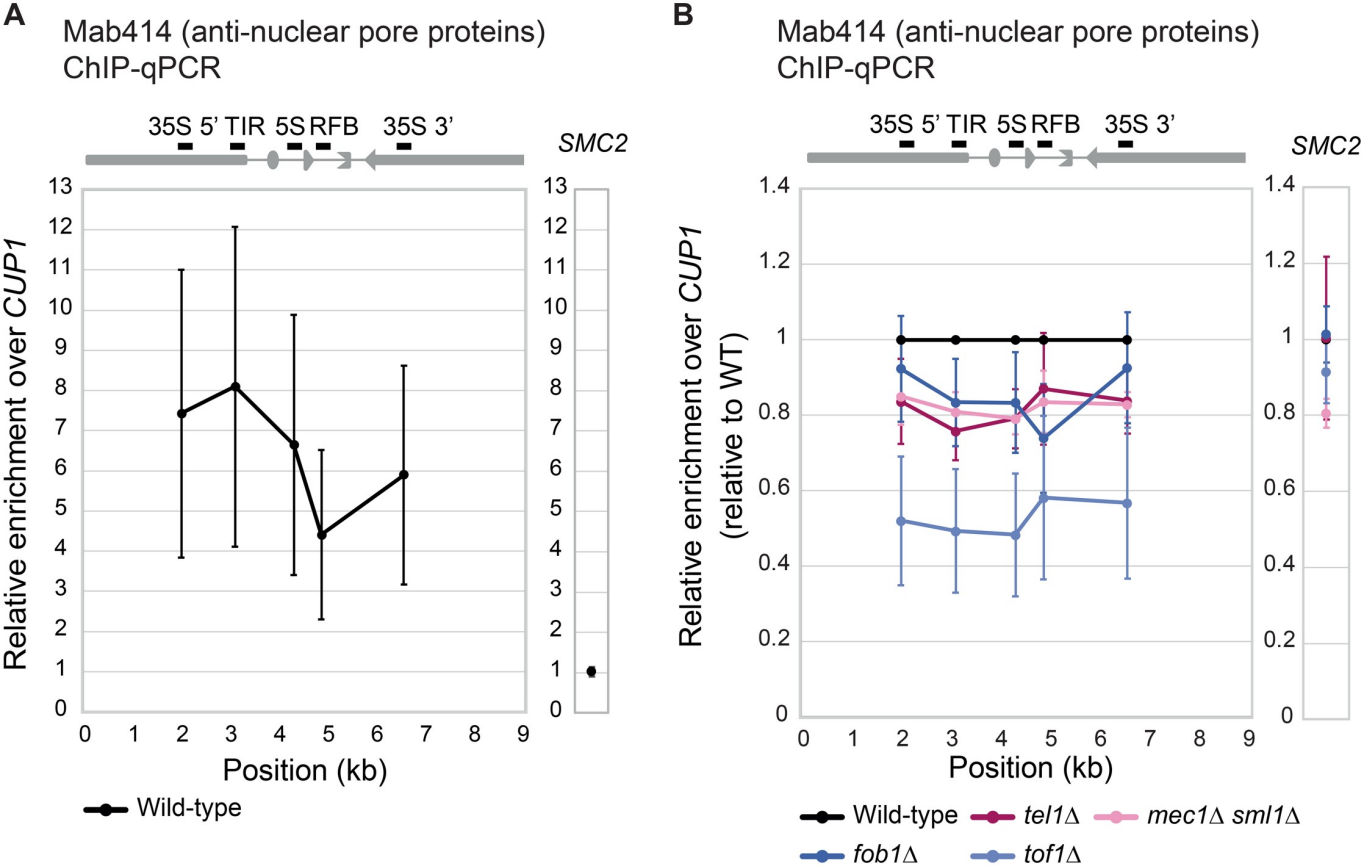
ChIP assay for rDNA-nuclear pore associations evaluated by real-time PCR. (A) Upper panel: Schematic drawing of five primer positions. Lower panel: ChIP assay for rDNA-nuclear pore was performed in the wild-type cells and quantified with the real-time PCR. Amount of precipitated DNA (rDNA and *SMC2*) relative to the *CUP1* locus. The error bars show the standard error of the mean (SEM) of three independent experiments. (B) To evaluate the difference between wild-type and mutant strains (*tel1*Δ, *mec1*Δ *sml1*Δ, *fob1*Δ and *tof1*Δ), we calculated the ratio of relative enrichment in wild-type and mutant strains in each experiment and compared the mean of three independent ChIP assays. *P*-values are shown in S2A Table. Error bars show the standard error of the mean (SEM) of three independent experiments.

Although the frequency of DSB was not increased in *tel1*Δ compared to wild-type, the mutant exhibited Fob1-dependent rDNA instability (Figs 2 and 1, respectively). A previous study demonstrated that Tel1 is required for translocation of HO-induced persistent DSBs to the nuclear pore and pore-binding is implicated in alternative recombination-mediated repair pathways [17]. Therefore, we hypothesized that replication-dependent DNA damage in rDNA might be associated with nuclear pores in a Tel1-dependent manner. To test this hypothesis, we performed chromatin immunoprecipitation (ChIP) assays with mAB414, which is an anti-nucleoporin antibody [20]. Five PCR primer sets in an rDNA unit were designed to detect precipitated rDNA, while two primer sets in *SMC2* and *CUP1* were used to detect control loci (Fig 3A). The precipitated rDNA was assessed by quantitative real-time PCR (qPCR) and relative enrichment was normalized against *CUP1*. Our results show that rDNA is enriched at the nucleoporins, which constitute nuclear pores, by 4.4- to 8.1-fold relative to the *CUP1* locus. By contrast, the control *SMC2* locus did not display any enrichment (Fig 3A). Intriguingly, enrichment immediately adjacent to the RFB was relatively weak by comparison to the surrounding regions (Fig 3A and S2A Table). Similar results were observed for the HO induced-DSB [17]. Although the underlying mechanism remains unclear, it may involve phosphorylation of histone H2A, recruitment of DNA repair proteins and/or DSB end resection around the DSB.

To evaluate the differences between wild-type and mutant strains, we calculated the relative enrichment of mutant strains to wild-type in each ChIP assay and compared the means of three independent assays (Fig 3B and S2A Table). The rDNA association with nuclear pores was significantly reduced both in *tel1*Δ and *mec1*Δ *sml1*Δ, suggesting that association of rDNA with the nuclear pores is dependent on DNA damage checkpoint kinases Tel1 and Mec1.

Tof1 is a component of the replisome and, like Fob1, is required for the arrest of the replication fork at the RFB and the formation of a DSB [24, 25]. To test whether the nuclear-pore association depends on the replication block in the rDNA, we performed the ChIP assay with the *fob1*Δ and *tof1*Δ, both of which do not exhibit the replication fork block at the RFB [8, 24, 26]. In the absence of Tof1, rDNA association with the nuclear pores was significantly reduced (Fig 3B and S2A Table). In contrast, the reduction was smaller for the *fob1*Δ and was not statistically significant. The reason for the observed differences between these two mutants is unclear. One possible explanation is that Fob1 is responsible for RFB only, while Tof1 might be related to replication fork arrest at any sites in rDNA as it travels with the replication fork. Indeed, there was no difference in binding to the nuclear pore at RFB between the *fob1*Δ and *tof1*Δ mutants (*P*-value = 0.303477. S2A Table). For *tof1*Δ, however, nuclear pore-binding was significantly decreased at non-RFB loci in rDNA (*P*-value < 0.05), except at the 3' end of 35S rDNA (*P*-value = 0.050003). This observation suggests, unlike Fob1, the role of Tof1 in nuclear pore binding is not limited to RFB sites (see Discussion section).

Because the replication fork block induces DNA damage only in S-phase, the association was expected to occur in this phase of the cell cycle. To confirm that, we synchronized cells in G1 phase and tested the association. Contrary to our expectation, the nuclear-pore association was detected even in G1 phase (S3 Fig). This raises the possibility that the association may be maintained throughout mitosis (see Discussion).

**Fig 4:**
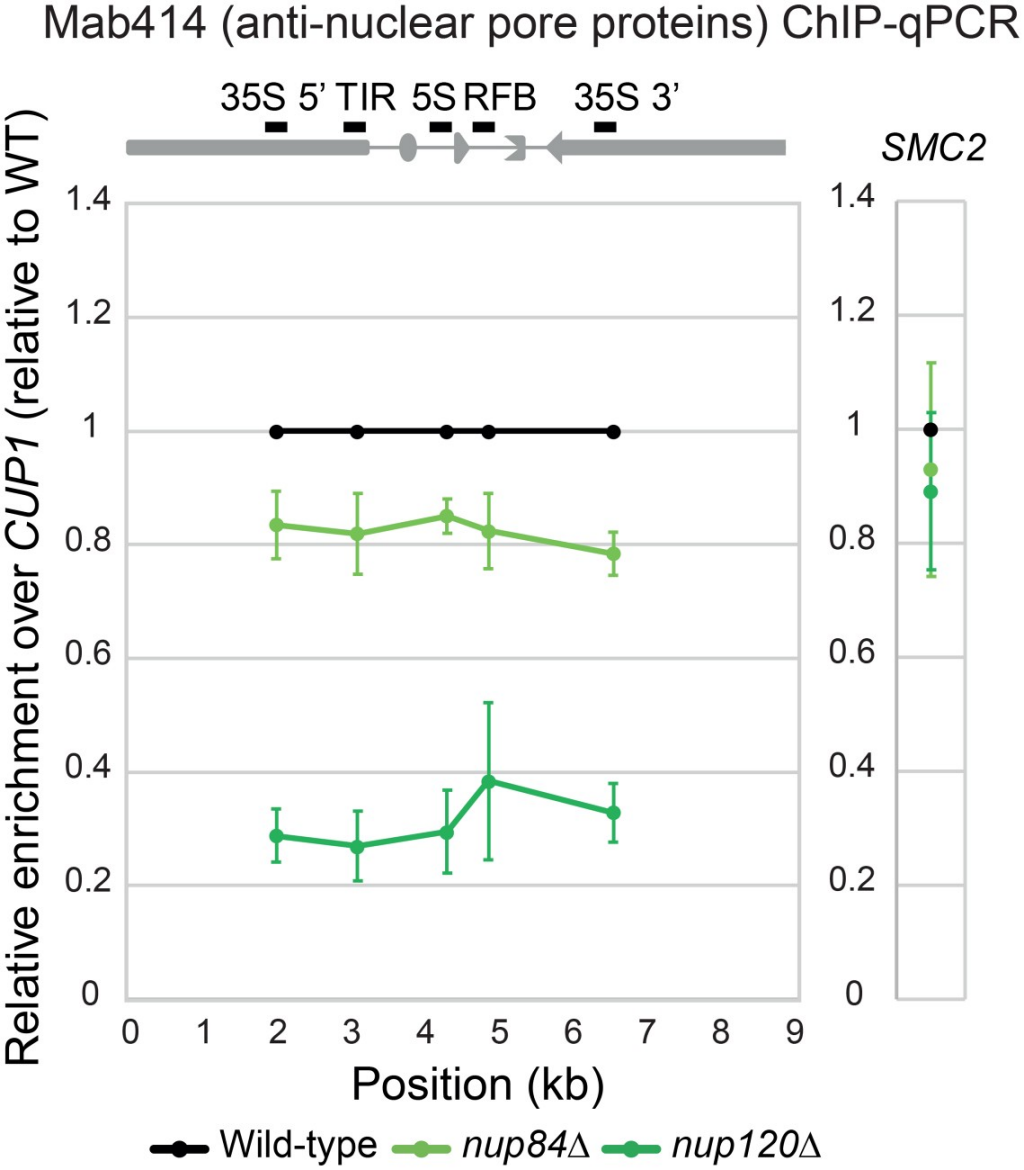
ChIP assay for rDNA-nuclear pore associations in the wild-type, *nup84Δ* and *nup120Δ* cells. The ChIP assay using quantitative PCR was performed as in Fig 3 for *nup84*Δ and *nup120*Δ. The error bars show the standard error of the mean (SEM) of three independent experiments. Because we performed the ChIP-qPCR experiment together with the ChIP assay shown in Fig 3, the same value of wild-type was used for computing the ratio.

In budding yeast, persistent DNA damage is recruited to the nuclear periphery and is associated with nuclear pores through the Nup84 subcomplex [17], which contains Nup133, Nup120, Nup145C, Nup85, Nup84, Seh1, and Sec13 [27–29]. The nuclear pore association of rDNA compromised both the *nup84*Δ and *nup120*Δ and the effect was more pronounced in the deletion of *NUP120*, suggesting that rDNA association with nuclear pores requires intact Nup84 complex (Fig 4 and S2A Table).

### Condensin recruiters Tof2, Csm1 and Lrs4 are required for rDNA-nuclear pore association

**Fig 5:**
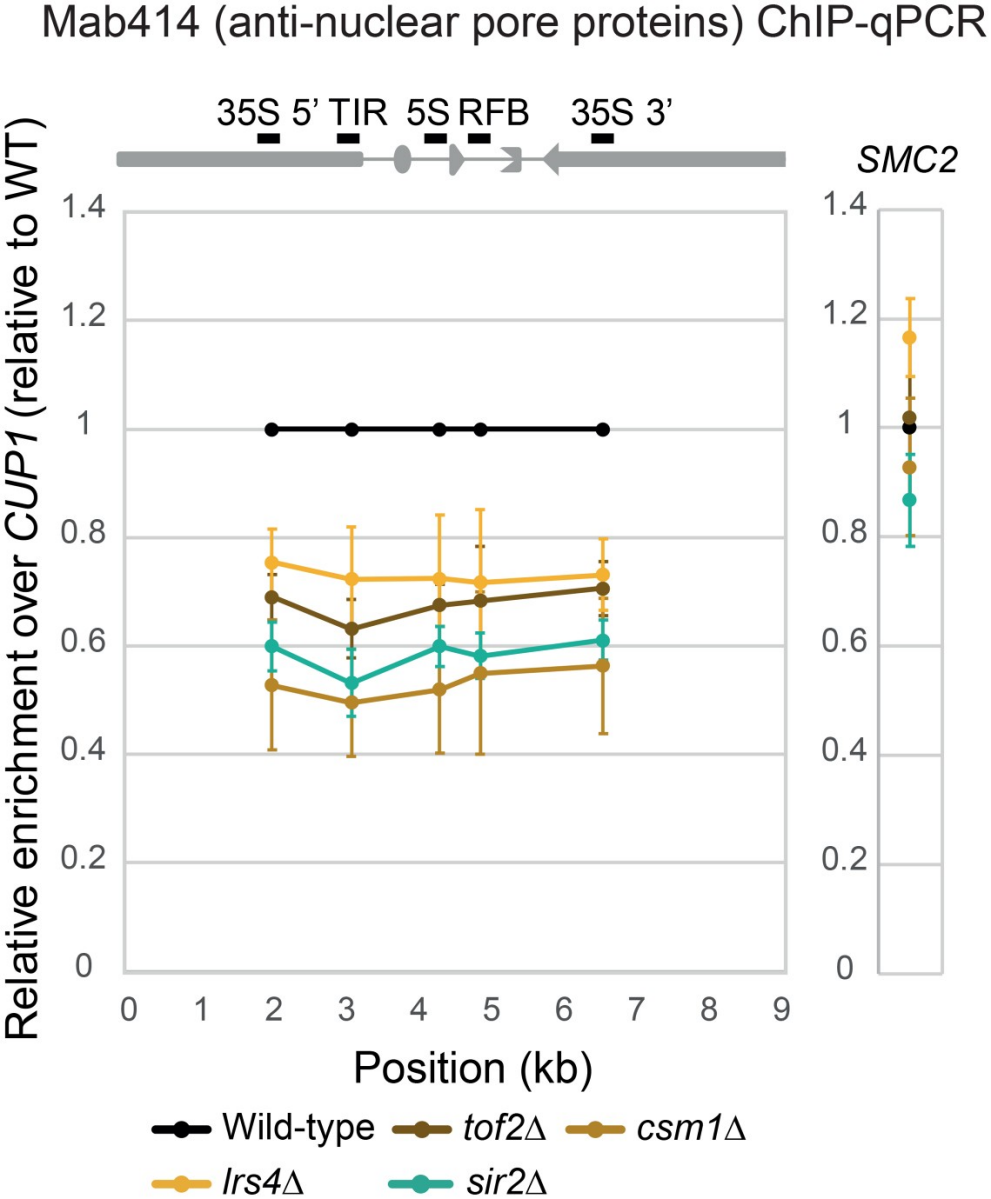
ChIP assay for rDNA-nuclear pore associations in the wild-type, *tof2*Δ, *csm1*Δ, *lrs4*Δ and *sir2*Δ mutant cells. The ChIP assay using quantitative PCR was performed as in Fig 3 for the *tof2*Δ, *csm1*Δ, *lrs4*Δ and *sir2*Δ mutants. The error bars show the standard error of the mean (SEM) of three independent experiments. Because we performed the ChIP-qPCR experiment together with the ChIP assay shown in Fig 3, the same value of wild-type was used for computing the ratio.

The rDNA gives rise to the nucleolus, which is a membrane-less organelle that appears to assemble through phase separation. Importantly, recombination foci are excluded from the nucleolus indicating that rDNA repair occurs in a specific environment distinct from the nucleolus [30]. Although Mec1/Tel1 have been implicated in nuclear pore association of DSB, there may be rDNA-specific factors that are involved in the nuclear pore association. We speculated that putative candidates would interact both with rDNA and with the nuclear pores or the surrounding nuclear membrane proteins. This holds for condensin recruiters Tof2, Csm1 and Lrs4, which have been identified as synthetic lethal mutants with a condensin conditional mutant (*smc2-157*) and that interact with Fob1 and recruit condensin to the rDNA [31]. Csm1 and Lrs4 are also known as cohibin that associates with CLIP (chromosome linkage inner nuclear membrane proteins, Src1 and Nur1) and localizes the rDNA to the CLIP to maintain rDNA stability, even though it has not been shown whether the binding is damage-dependent [32, 33]. To test the contribution of these proteins to the association of rDNA with nuclear pores, we performed a ChIP-qPCR assay with deletion mutants for the factors. The rDNA association with the nuclear pores in all these mutants was reduced compared to wild-type, indicating that condensin recruiters are required for rDNA relocation to the nuclear pores (Fig 5 and S2A Table). Sir2 also acts as a bridge between rDNA and the nuclear pores as is the case for CLIP (Fig 5 and [32]). For *sir2*Δ, the association of rDNA with the nuclear pores was also reduced (Fig 5 and S2A Table).

### I-*Sce*I induced DSB in the rDNA is localized to the nuclear periphery

**Fig 6:**
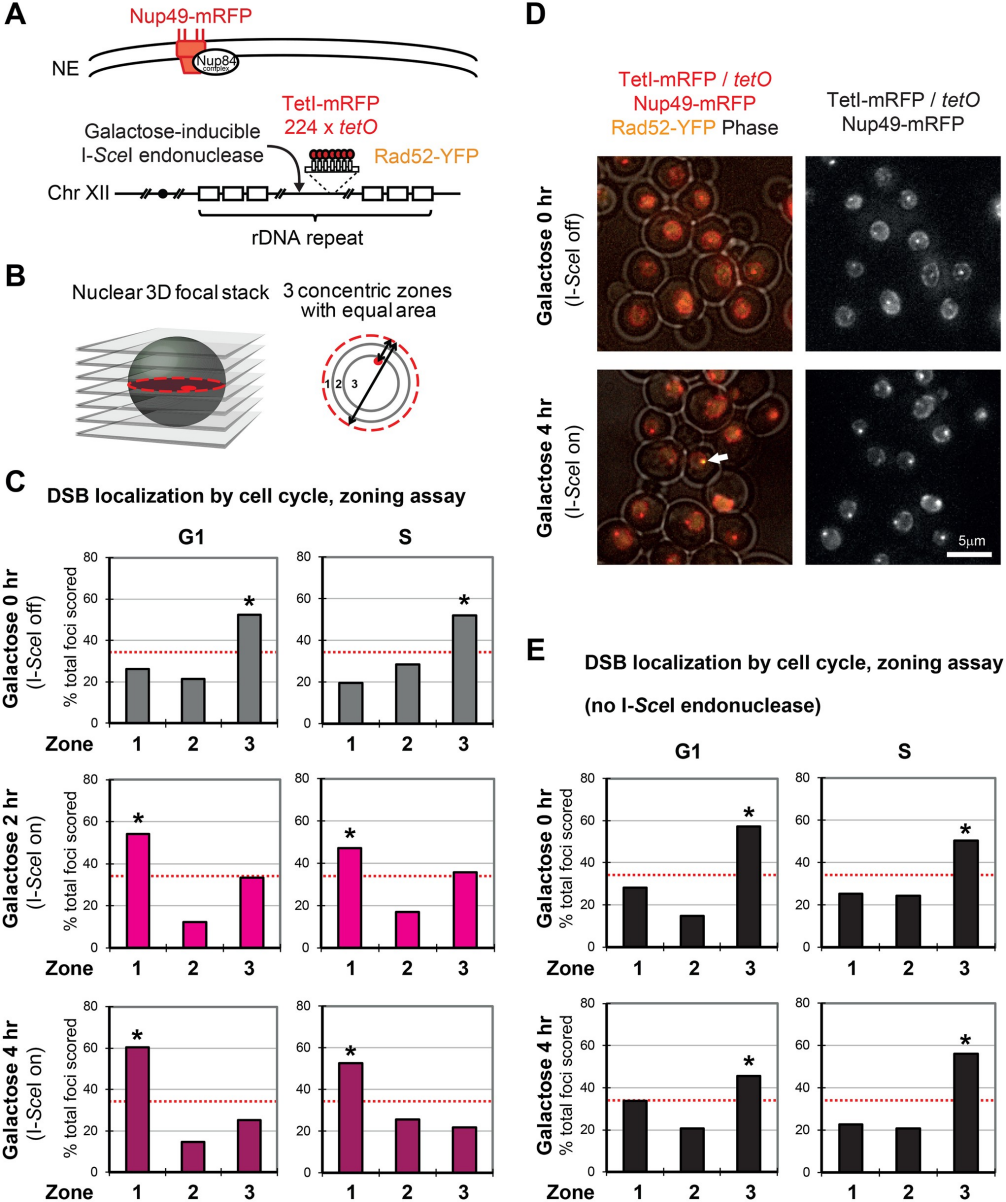
Localization of an I-*Sce*I-induced DSB in the rDNA. (A) Illustration of the inducible DSB in rDNA and its visualization. An I-*Sce*I cut site and a *tetO* array were inserted into a site of the rDNA repeat [30]. TetI-mRFP and Nup49-mRFP label the position of I-*Sce*I cut site and nuclear pores, respectively. (B) Locus position was scored relative to the nuclear diameter in the locus’ plane of focus using an image stack. Distance over diameter ratios were binned into 3 equal zones. (C) Position of cleaved I-*Sce*I cut site in rDNA relative to Nup49-mRFP after 2 and 4 hours on galactose. The relocation to the nuclear periphery was observed in both G1 and S phase of wild-type cells. Although cleavage efficiency was calculated as 97% by real-time PCR, Rad52 positive cells were 20% of total cells at 4 hours after galactose addition (n = 285). For this reason, we scored the position of TetI-mRFP / *tetO* regardless of the presence or absence of Rad52 signal. Counted nuclei and statistical significance are indicated in S2C Table. (D) Representative images before and 4 hours after DSB induction are shown. The 3D stack images were projected to a 2D plane by standard deviation. The white arrow marks a Rad52-YFP focus colocalizing with TetI-mRFP signal on the cleaved rDNA. (E) Position of the I-*Sce*I cut site in rDNA relative to Nup49-mRFP in the cells not expressing I-*Sce*I. * = significantly non-random based on cell number and confidence values from a proportional test comparing random and experimental distributions. Red dotted line indicates 33% or random distribution.

To determine the subnuclear localization of spontaneously damaged rDNA, we used a strain in which each copy of the rDNA repeat has a *lacO* array that associates with LacI-GFP [34]. We scored DSBs on the rDNA by monitoring the foci of Rad52-CFP, a factor essential for homologous recombination that accumulates at DSBs (S4A and S4B Fig). The Rad52 focus was barely detected under normal physiological conditions (4 cells scored from 875 asynchronous cells; 1.26%) and colocalization of Rad52-CFP and LacI-GFP / rDNA-*lacO* was even less frequent (0.46%). Note that Rad52 foci are formed only when the DSBs are excluded from the nucleolus [30] and we estimate that less than 21% of DSBs are marked by discrete Rad52 foci in the rDNA (see legend to Fig 6C). This may result in a loss of data for a large fraction of DSBs if we use Rad52 as a marker of DSB in the rDNA.

Instead, we used I-*Sce*I endonuclease to induce DSB in the rDNA [30]. In this assay, I-*Sce*I cleaves the recognition sequence inserted in the rDNA and the location of DSB is detected by TetI fused with mRFP (monomeric red fluorescent protein) that associates with the adjacently located *tetO* array [30] (Fig 6A). The I-*Sce*I induced DSB is known to shift away from the nucleolus to complete homologous recombinational repair [30]. Using this system, we scanned the position of the TetI-mRFP focus and classified them into three zones compared with mRFP-fused nuclear pore proteins [35] (Fig 6B). Before induction of I-*Sce*I, the TetI-mRFP locus was preferentially positioned in the nuclear center. Strikingly, the locus was relocated to the nuclear periphery both in the G1 and S phases within 2 hours of DSB induction (Fig 6C and 6D). No enrichment was observed in the strain lacking the I-*Sce*I endonuclease, confirming the association is damage-specific (Fig 6E). These results indicate that DSB in the rDNA is localized in the nuclear periphery.

### The nuclear-pore association of rDNA is important for its stability

**Fig 7:**
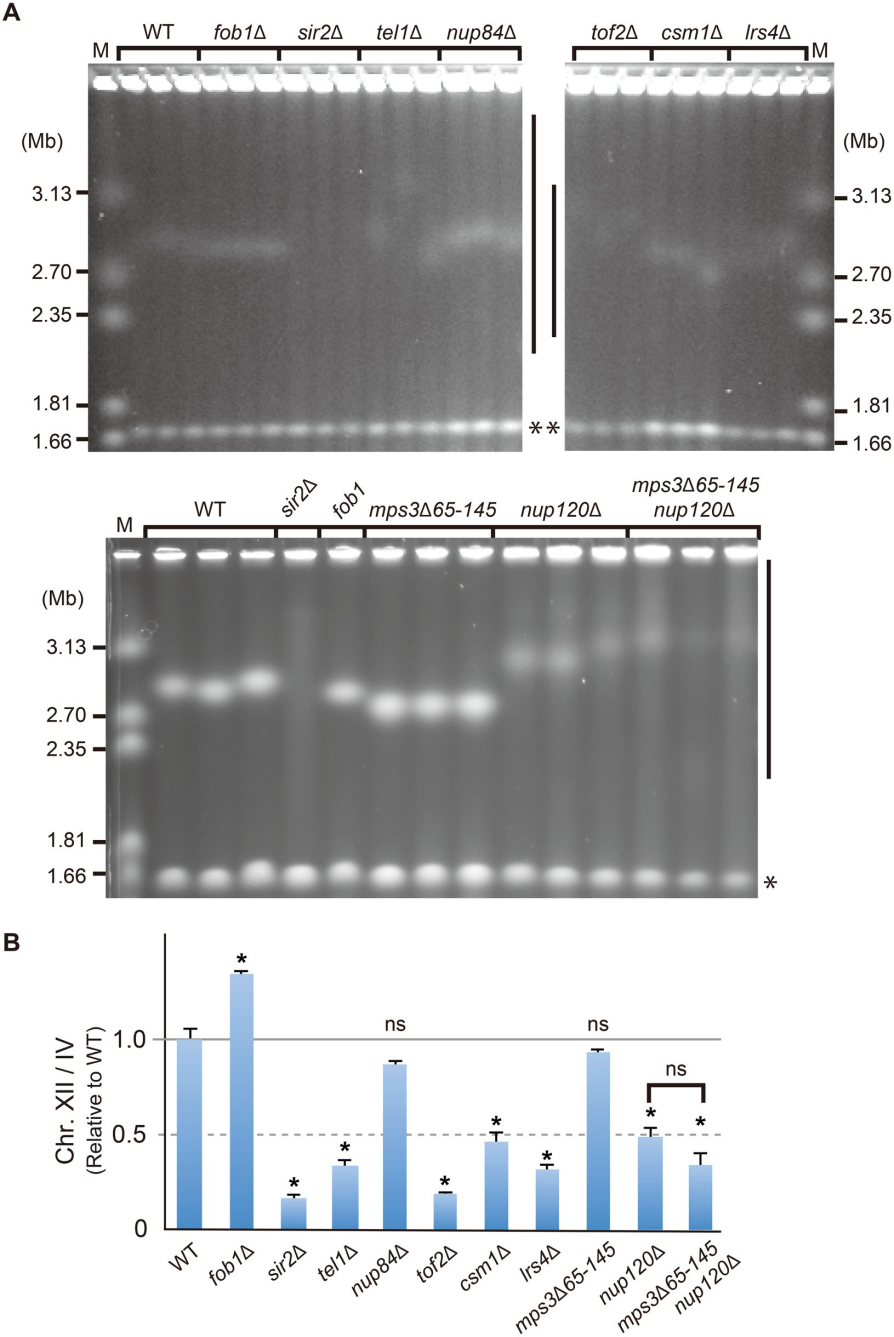
rDNA stability in rDNA-nuclear pore associations defective mutants. (A) Pulse field gel electrophoresis was performed, and the gels were stained with ethidium bromide (EtBr) for visualization. The two largest chromosomes (XII and IV) are shown. Side bars and asterisks mark the position of chromosome XII and IV, respectively. M is the size marker (*H*. *wingei* chromosomes). (B) Quantitation of rDNA instability shown in A. Signal intensities were quantified as Fig 1C (see Materials and methods). The values are relative to that in the wild-type strain. Error bars show the standard error (SEM) of three independent colonies. The significance levels (* *p* < 0.05) are from the unpaired two-tailed *t*-tests. ns, not significant. *P*-values are shown in S2B Table.

To test whether rDNA association with the nuclear pores has a biological role in maintaining rDNA stability, we analyzed the migration of chromosome XII in mutants that fail to relocate rDNA to the nuclear pores (*sir2*Δ, *tel1*Δ, *nup84*Δ, *nup120*Δ, *tof2*Δ, *csm1*Δ, and *lrs4*Δ) by pulsed field gel electrophoresis (PFGE, Fig 7A and 7B). The *fob1*Δ and *sir2*Δ were used as the negative and the positive control, respectively. All mutants except for *nup84*Δ exhibited an unstable chromosome XII compared to the wild-type (Fig 7A and 7B). Nup84 and Nup120 belong to the same heptameric Nup84 complex of nuclear pore complex [28, 29, 36]. However, the nuclear pore association and the stability of rDNA were differentially affected in these mutants (Figs 4 and 7). These findings are consistent with the fact that DNA damage sensitivity in the *nup120*Δ is stronger than that in the *nup84*Δ [37]. Taken together, these data suggest that Nup120 plays a more prominent role than Nup84 in DNA repair through an unknown mechanism.

Mps3 acts as an alternative anchoring site of HO-induced DSBs on the nuclear membrane [18, 19, 38]. A mutant form of the essential Mps3 (*mps3*Δ*65–145*), truncated at the N-terminal acidic domain, did not affect rDNA stability according to PFGE analysis (Fig 7A and 7B, [39]). Furthermore, *nup120*Δ *mps3*Δ*65–145* double mutations did not show any additive effect in terms of rDNA-stability compared to the corresponding single mutations, suggesting that Mps3 does not make a significant contribution to rDNA stability. Given that rDNA instability in *tel1*Δ was dependent on Fob1 (Fig 1B and 1C), the replication-dependent DNA damage in rDNA appears to bind to the nuclear pores for its maintenance.

## Discussion

rDNA is one of the most unstable regions in the genome due to its repetitive nature. Recombination among the repeats would result in deletions (loss of copies) leading to copy number instability. Nonetheless, cells appear to have evolved mechanisms to avoid such instability, which would be deleterious. Association of rDNA to the nuclear pores seems to be one such mechanism. By this change in location, the broken rDNA unit is isolated from intact copies and the risk of hazardous recombination thereby reduced. Moreover, alternative repair pathways at the nuclear pore might be facilitated [17, 40].

**Fig 8:**
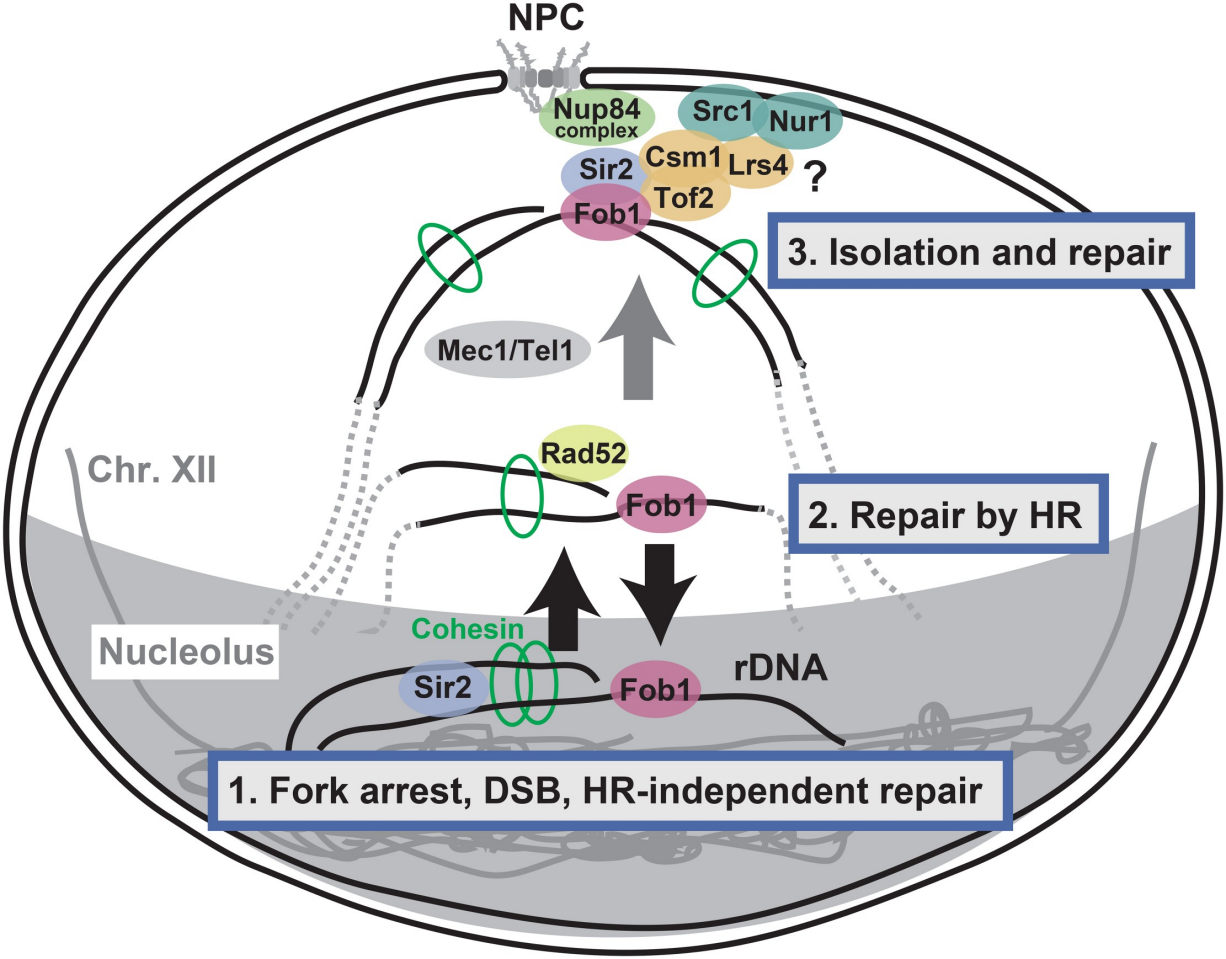
Model of a multistage process of rDNA repair. There are three stages of rDNA repair. See the text for details. The outer oval shows the nuclear envelope of a budding yeast cell. The lower gray area represents the nucleolus. Solid and dotted lines indicate double-stranded DNA. NPC: nuclear pore complex. For clarity, DSB in the rDNA are shown to localize with nuclear pores that are distant from the nucleolus. However, in reality, the nuclear pore-rDNA binding site could be close to the nucleolus. Question mark: It has been shown that Csm1 and Lrs4 connects Sir2 on rDNA to nuclear membrane proteins, Src1 and Nur1 [32]. Moreover, these proteins play a significant role in rDNA-binding to the nuclear pore and maintaining its stability (Figs 5 and 7). Nonetheless, the perinuclear protein bridge might not have an effect on the binding of damaged rDNA to the nuclear pore.

In Fig 8, we summarize how the damaged rDNA is repaired. Recently, we found that the ends of a DSB formed after stalling of a replication fork at the RFB are not resected in a strain with a normal rDNA copy number, and that the DSB is repaired through a pathway that does not involve homologous recombination [9]. In this pathway, the DSB can be repaired without alteration of rDNA copy number. Therefore, we proposed that this homologous recombination-independent repair is the default mechanism used for rDNA repair (1st stage, Fig 8). In contrast, when the rDNA copy number is reduced in a strain, resection of the DSB is induced, which triggers unequal sister-chromatid recombination that may amplify the number of rDNA copies [9]. For this reaction, the DSB together with the surrounding region needs to be moved from the nucleolus to the nucleoplasm where the homologous recombination enzymes, including Rad52, form distinct foci (2nd stage) [30]. Previously, we found that E-pro transcription is activated and cohesin dissociates from the rDNA in the absence of Sir2. As a result, unequal sister-chromatid recombination was increased and the copy number changed with a high frequency [10] (S1 Fig). The E-pro regulated recombination may occur at this stage just outside of the nucleolus. Finally, if the DSB cannot properly be repaired at the 2nd stage, the DSB with the surrounding region relocates to the nuclear envelope where it is trapped by the nuclear pores (3rd stage). In the presence of a repair template, no binding of the DSB to the nuclear periphery was observed in a previous HO-induced DSB assay [17, 19]. Although there are abundant repair templates in the case of damaged rDNA, the locus is relocated to the nuclear pores presumably because it is isolated from the majority of templates at the 2nd and 3rd stages. The 3rd stage may work as a back-up system for the 1st and the 2nd stages and could prevent aberrant genomic changes such as the generation of a large deletion. The isolated broken ends around the nuclear pores may be repaired by homologous recombination with chromosomal rDNA or an ERC. Otherwise, repair of the broken ends may occur via the single strand annealing (SSA) pathway that connects repetitive sequences using the homologous sequence without introducing mutations [41]. In this study, proteins involving replication fork bock, DNA damage checkpoint and condensing loading were implicated in the rDNA-nuclear pore binding. Unraveling the hierarchy of these factors is an exciting challenge for future studies.

In the *tof1*Δ, defects in the association to the nuclear pores were more obvious than in the *fob1*Δ (Fig 3B). The reason for the difference in dissociation between these mutants is unclear. One possible explanation is that Fob1 is specifically responsible for the RFB, while Tof1 might be associated with replication fork arrest at any site in rDNA given that it travels with the replication fork. In the *fob1* mutant with a low rDNA copy number, collision between 35S transcription and replication machineries causes inhibition of the replication fork and induces rDNA instability [42]. This damage to the DNA may occur to some extent in a normal copy strain and trigger the relocation. By contrast, in the *tof1* mutant, such RFB independent damage might also be reduced, resulting in a lower level of nuclear pore binding.

The binding of rDNA to nuclear pores was detected even in the G1 phase (S3 Fig). Because no replication-dependent DSB is induced in G1 phase, the data does not easily fit the DSB dependent-binding model (Fig 8). Nonetheless, there are several possible explanations for the cell cycle independent association of rDNA to nuclear pores. The first interpretation is that the binding is caused by extra-chromosomal rDNA circles (ERCs) that are produced by unequal sister chromatid recombination. However, the ChIP data in *sir2*Δ does not support this hypothesis (Fig 5). Because *sir2*Δ leads to instability of rDNA and produces vast amounts of ERCs, the strains should show an accumulation of rDNA-nuclear pore binding if ERCs bind to the nuclear pores. However, no such accumulation was observed. An alternative interpretation is that a DSB in rDNA that is not repaired in S/G2 phases might be carried into the next cell cycle. It is known that damage in the rDNA does not induce checkpoint control [43]. Once a DSB in rDNA is carried over to the next cell cycle, it can be recruited to or maintained at the nuclear periphery in G1 phase as seen in endonuclease-induced damage (Fig 6). A third interpretation of cell-cycle independent interaction of rDNA to nuclear pores is that the rDNA binds to the nuclear pore and is maintained at the site even after repair is completed. The replication-dependent rDNA damage occurs in S-phase and rDNA is relocated to the nuclear periphery. The DSB in rDNA is repaired in S/G2 phases and the locus might be kept at the nuclear periphery until the next G1 phase. In either case, we hypothesize that a small portion of damaged rDNA remains in the mother cell with the nuclear envelope, which may be carried into the next cell cycle. Indeed, we detected stacked rDNA in the wells during pulse-field gel electrophoresis specifically of mother-cells in G1 phase (three or four budded age). This observation suggests an accumulation of unstable rDNA in the G1 phase of mother cells [44]. We propose that this accumulation of broken ends could be a cause for senescence of the mother cell.

Several recent papers highlight the importance of perinuclear anchoring for continuing damage repair. It has been shown that replication damage associated with expanded triplet repeats and eroded telomeres shift transiently to the nuclear pores [45, 46]. Su *et al*. showed that an artificially inserted CAG repeat is localized to the nuclear pores in a replication-dependent manner and this localization was important for CAG repeat stability [45]. As the repeat may form a secondary structure and arrest replication, the CAG repeats and rDNA are expected to share a common mechanism that localizes them to the nuclear periphery, at least partially. Churikov *et al*. showed that shortened telomeres in a telomerase-deficient yeast strain are relocated to the nuclear pores and this localization was required for type II survivors in which the short terminal TG-tract is elongated by recombination (ALT in mammals) [46]. Although the relationship between the shortened telomere recombination and rDNA stability is not known, localization at the nuclear pore seems to be important for many aspects of genome maintenance.

In this study, we identified a mechanism that protects damaged repetitive rDNA sequences from undergoing rearrangement (copy number variation) by association with the nuclear pores. In this way rDNA stability is maintained probably via the SSA pathway, which cannot be applied to DSBs in non-repetitive sequences. Likewise, in *Drosophila* cells, a DSB in heterochromatin that mostly comprises repetitive sequences relocates to the nuclear pores for repair in a SUMOylation-dependent manner [47]. SUMOylation also mediates relocation of the DSB in the rDNA to outside of the nucleolus and the eroded telomere to the nuclear periphery in *Saccharomyces cerevisiae* [30, 46]. It has been reported that damaged rDNA is relocated to specific loci around the nucleolus of mammalian cells and most of the factors required for this relocation, which were identified in yeast, are well conserved [48]. Because mammalian genomes contain large stretches of repetitive sequences, such as retrotransposons and Alu-repeats, a similar mechanism may operate to maintain genome integrity in higher eukaryotes. Future studies will shed light on the involvement of human homologues in the repair of damaged repetitive DNA.

## Materials and methods

### Yeast strains, primers and growth conditions

Yeast strains used in this study were derived from NOY408-1b (a W303 derivative). Strains were grown at 30°C in YPD (YPDA for Figs 1, 3, 4, 5, 7 and S3 Fig) medium. YPD (yeast extract-peptone-dextrose) and YPDA (YPD with 0.4% adenine) are rich media used for normal culture. Synthetic complete (SC) medium lacking the appropriate amino acids [49] was used for gene marker selection. Yeast strains used in this study are listed in S1 Table. If necessary, G418 (Sigma) or clonNAT (WERNER) was added to the medium at the following concentration, 500 μg/ml (G418) or 100 μg/ml (clonNAT). Yeast genetic transformation was performed by using Frozen-EZ Yeast Transformation II Kit (Zymo Research Corporation) according to the manufacturer’s instructions. To test rDNA stability by pulsed field gel electrophoresis, we used cells that had divided ~45 times after transformation.

For the DSB localization assay, yeast cells were grown at 30 °C for 2 days on selective synthetic medium containing 2% glucose (SD). The cells were inoculated in synthetic medium containing 2% raffinose (SR) and grown overnight. The culture was diluted to SR next morning and grown for about 4 hours. When the exponentially growing cell population reached around 2.5 × 10^6^ cells ml^−1^, we added 20% galactose (final 2%) to the medium to induce I-*Sce*I. The living cells were directly subjected to microscopy on an SR agarose pad. We used SD/SR-lacking tryptophan and uracil for YCH-252 or lacking tryptophan, uracil and histidine for YCH-244 in these experiments.

### Pulsed-field gel electrophoresis

Samples for pulsed-field gel electrophoresis (PFGE) were prepared as described previously [50]. Electrophoresis was performed in a 1% (0.8% for S2B Fig) agarose gel with 0.5×Tris-borate-EDTA (TBE) buffer, using CHEF-MAPPER (Bio-Rad). The conditions were a 300–900 sec pulse time and 100 V for 68 hours at 14 °C. For S2B Fig, after electrophoresis, the rDNA was detected by Southern blot analysis with an rDNA specific probe. To quantify instability of rDNA in PFGE (Figs 1C and 7B), the signal intensities of Chr. XII and Chr. IV were measured by Image J (Fiji) using the image of an EtBr stained gel. The signal intensities of Chr. XII were divided by that of Chr. IV, which was expected to be constant between mutants. Broader unstable bands reduce signal intensities in the area. Moreover, chromosomes with an unusual structure cannot enter the gel and thereby reduce signal intensity. Normalization of the Chr. XII band intensity in the mutants to that of Chr. IV, yielded values reflecting their rDNA stability. In the *tof2*, *csm1* and *lrs4* mutants, several minor bands were observed. This suggests some of the cells contained multiple copies of chromosome XII because of chromosome missegregation caused by condensation defects in these mutants [31]. In such cases, the major band was measured.

### Two-dimensional (2D) gel electrophoresis

2D gel electrophoresis was performed as previously described [51]. DNA from early log phase cells (~3x10^6^ cells/ml in YPD medium) were digested in agarose plugs (5x10^7^ cells/plug) using *Bgl*II for 4 h at 37 °C. The reaction was carried out in 200 μl reaction buffer with 150 units of *Bgl*II. After electrophoresis, the rDNA was detected by Southern analysis with an rDNA specific probe. RFB and DSB signals were quantified by ImageQuant (GE). The signal intensity of the RFB spot was divided by the signal intensity of total replication intermediates signal for normalization. The signal intensity of the DSB spot was normalized to the RFB signal to show the relationship between the DSB and the arrested fork it was derived from.

### Chromatin immunoprecipitation evaluated with quantitative real-time PCR (ChIP-qPCR)

ChIP was carried out as previously described [52] with minor modifications described below. Yeast cells cultured in 45 ml medium were cross-linked with 1% formaldehyde at 30 °C for 20 min. Cell pellets were resuspended in 600 μl of lysis buffer (50 mM HEPES-KOH at pH 7.5, 500 mM NaCl, 1 mM EDTA at pH 8.0, 1% Triton X-100, 0.1% sodium deoxycholate and protease inhibitors) and disrupted with zirconia beads using a Multi-bead shocker (Yasui Kikai). The recovered chromatin fraction was subjected to sonication using a Bioruptor (Cosmo Bio) to obtain fragmented chromatin < 500 bp in length. An anti-nuclear pore FG-repeat antibody (mAB414, Abcam) combined with Dynabeads Protein G (Thermo Fisher), was used for IP. Beads were washed twice in lysis buffer, once with wash buffer (10 mM Tris-HCl at pH 8.0, 250 mM LiCl, 0.5% Nonidet P40 (IGEPAL), 0.5% sodium deoxycholate, 1 mM EDTA at pH 8.0 and protease inhibitors), and once with TE (10 mM Tris-HCl at pH 8.0 and 1 mM EDTA at pH 8.0) at 4 °C. ChIP DNA was purified and analyzed by quantitative real-time PCR using primers amplifying various regions of the rDNA, the *SMC2* (condensin complex) locus on Chr. VI or the *CUP1* locus on Chr. VIII (primer sequences are listed in S3 Table). Enrichment was normalized to that from the genomic *CUP1* locus in IP and Input DNA samples and were calculated as [rDNA or *SMC2* (IP) / *CUP1* (IP)] / [rDNA or *SMC2* (Input) / *CUP1* (Input)]. Details of the formula used for these calculations is given below:

Relativeenrichment=%Input(Testlocus)/%Input(Controllocus)

%Input(Testlocus)=100×2∧(Ct(AdjustedInput)−Ct(IP))

%Input(Controllocus)=100×2∧(Ct(AdjustedInput)−Ct(IP))

Ct(AdjustedInput)=Ct(Input)−LOG(10,2)

The “Relative enrichment over *CUP1*” is shown in Fig 3A. To compare wild-type and mutant cells, we divided the values corresponding to the mutants (or G1-phase wild-type cells in S3 Fig) by that of the wild-type cells (or asynchronous wild-type cells in S3 Fig) in each ChIP assay. The mean values of three (Figs 3, 4 and 5) and five (S3 Fig) independent assays are shown.

### Microscopy and statistical analyses

Fluorescence microscopy and quantification was performed according to published methods [35, 53] using an ECLIPSE Ti microscope (Nikon) fitted with a Zyla 4.2P sCMOS (Andor Technology) camera. TetI-mRFP position was determined with a through-focus stack of 12 0.3 μm steps and was measured by ImageJ (Fiji) and the plug-in software PointPicker [53]. The numbers of nuclei scored are shown in S2C Table. The efficiency of DSB induction was determined by real-time PCR with SYBR Green as previously described [54]. To determine zone enrichment, we applied a χ^2^ test comparing zone 1 or zone 3 to a random distribution (degree of freedom = 2, confidence limit = 95%). *p*-values are indicated in S2C Table.

## Supporting information

S1 Fig Recombination in rDNA. rDNA recombination occurs in a RFB-dependent manner. The RFB site induces a DSB that is repaired by recombination between sister-chromatids. The repair is controlled by Sir2 and E-pro. When the rDNA copy number is reduced, E-pro transcription is activated, which prevents cohesin from associating to the surrounding regions. In this situation, recombination can occur unequally and the rDNA copy number increases (S1B-1 Fig) or an ERC is produced (S1B-2 Fig). When the copy number is at the wild-type level, Sir2 represses E-pro transcription and cohesin can associate, leading to equal sister-chromatid recombination that does not change the copy number (S1A Fig); thus, rDNA is stable. This figure is reproduced with authors’ permission from ref [12].
(AI) Click here for additional data file.

S2 Fig PFGE assays and Southern blotting. (A) Pulsed field gel electrophoresis for assessing rDNA stability in the *tel1*Δ and *tel1*Δ *fob1*Δ mutants. To increase the number of test transformants and trials, we repeated PFGE assays using six independent colonies. M is the size marker (*H*. *wingei* chromosomes). (B) Pulsed field gel electrophoresis for assessing rDNA stability in the *tel1*Δ mutant. Two independent transformants were tested. Left: the gel was stained with ethidium bromide (EtBr). The size marker is formed by *H*. *wingei* chromosomes. Right: the gel was analyzed by Southern blot analysis using an rDNA probe.
(PDF)

S3 Fig ChIP assay for rDNA-nuclear pore associations in G1-phase cells. ChIP assay for rDNA-nuclear pore associations in asynchronous and G1-arrested wild-type cells. The cells were arrested in G1-phase by α-factor treatment for 90 min. The ChIP assay using quantitative real-time PCR was performed as in Fig 3, whereas the assays were done independently of Fig 3. The error bars show the standard error of the mean (SEM) of five independent experiments.
(PDF)

S4 Fig Colocalization of Rad52-CFP and rDNA. (A) Colocalization of a Rad52-CFP, Nup49-mRFP and LacI-GFP / rDNA-*lacO*. A representative image is shown. A magnified window shows the colocalization of Rad52-CFP and rDNA. (B) Through-focus stack images of 12 0.3 μm steps were used to determine the colocalization. Rad52-CFP position was compared with LacI-GFP/ rDNA-*lacO* and Nup49-mRFP. We defined the following three situations as colocalization: fully overlapping, partially overlapping, and juxtaposition.
(PDF)

S1 Table List of yeast strains used in this study. (XLSX)

S2 Table Summary of statistics in this study. (A) *p*-values for ChIP assays. (B) *p*-value for 2D gel and PFGE assays. (C) Summary of localization assay statistics.
(XLSX)

S3 Table List of primer pairs used in ChIP assays. (XLSX)
