# Supplementary figures and images for: Ribosomal RNA gene repeats associate with the nuclear pore complex for maintenance after DNA damage
Source: PLoS Genet. 2019 Apr 18;15(4):e1008103. doi: 10.1371/journal.pgen.1008103 (PMC6490929; doi:10.1371/journal.pgen.1008103)

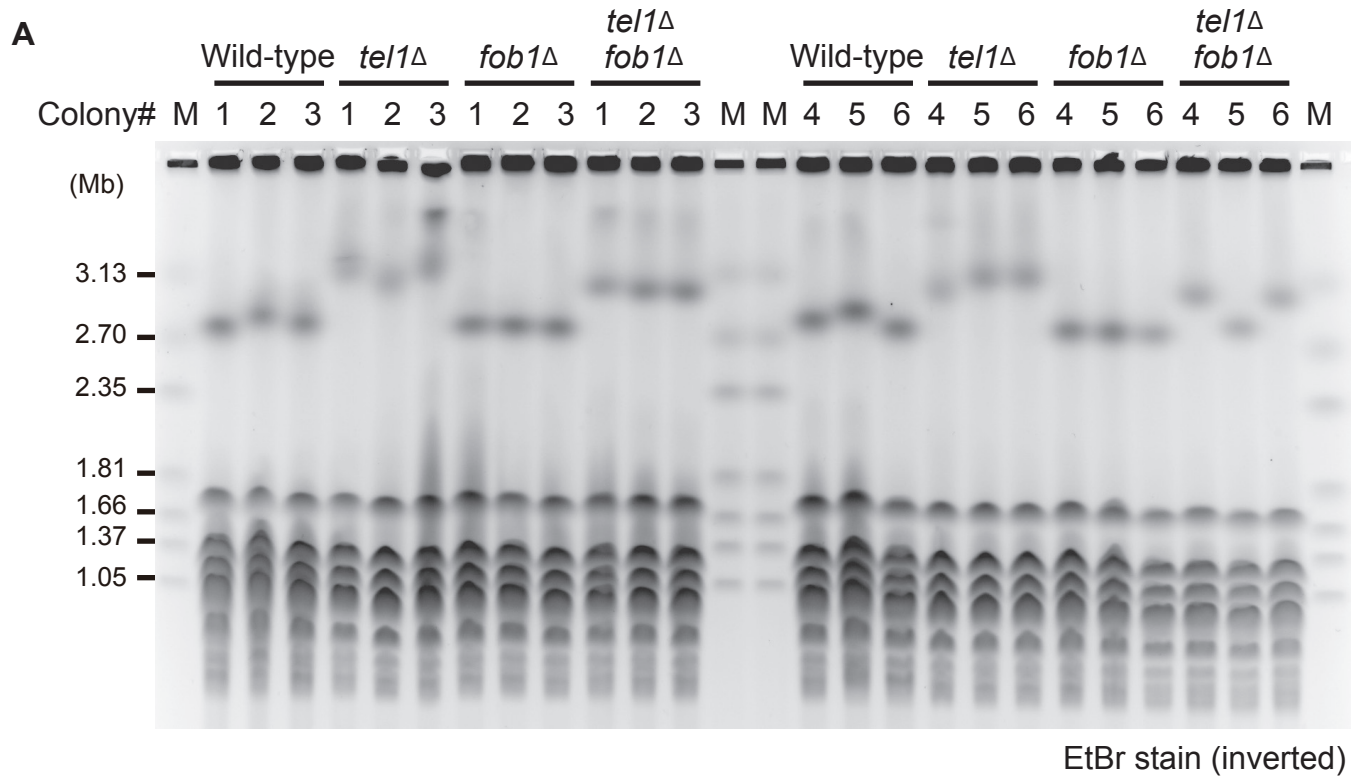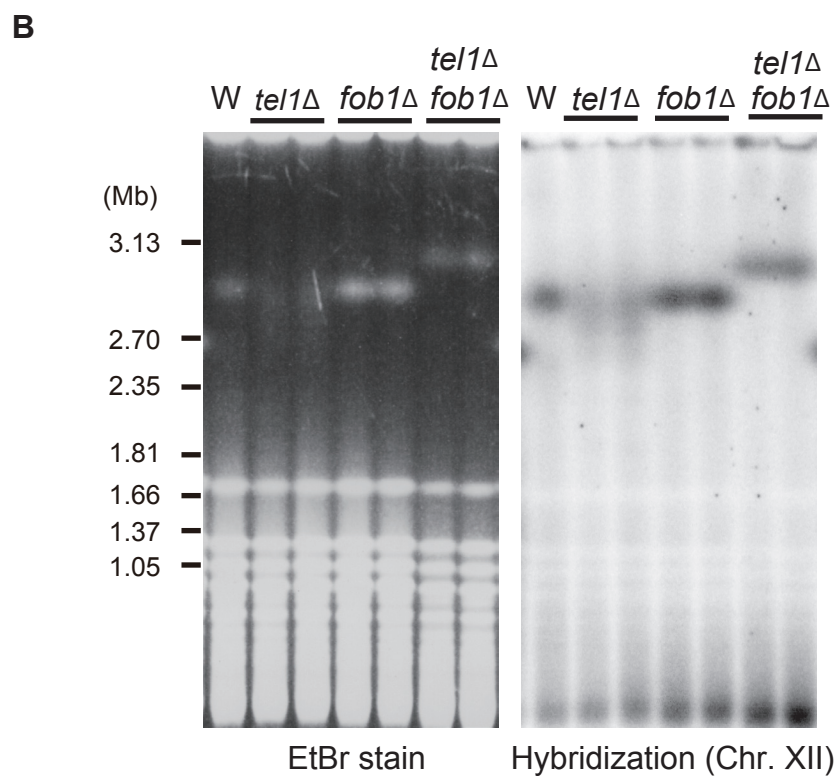

Supplement: S2 Fig — (A) Pulsed field gel electrophoresis for assessing rDNA stability in the tel1Δ and tel1Δ fob1Δ mutants. To increase the number of test transformants and trials, we repeated PFGE assays using six independent colonies. M is the size marker (H. wingei chromosomes). (B) Pulsed field gel electrophoresis for assessing rDNA stability in the tel1Δ mutant. Two independent transformants were tested. Left: the gel was stained with ethidium bromide (EtBr). The size marker is formed by H. wingei chromosomes. Right: the gel was analyzed by Southern blot analysis using an rDNA probe. (PDF) [file pgen.1008103.s002.pdf]

Mab414 (anti-nuclear pore proteins) ChIP-qPCR

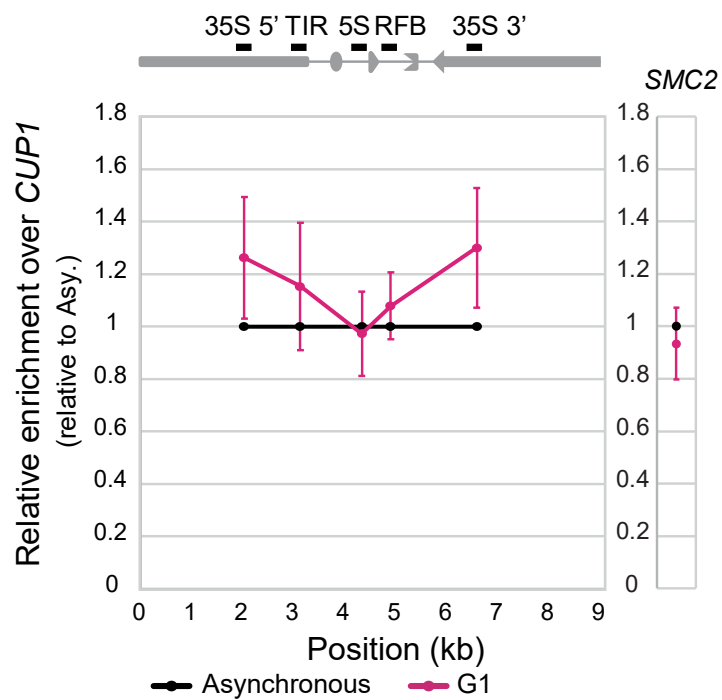

Supplement: S3 Fig — ChIP assay for rDNA-nuclear pore associations in asynchronous and G1-arrested wild-type cells. The cells were arrested in G1-phase by α-factor treatment for 90 min. The ChIP assay using quantitative real-time PCR was performed as in Fig 3, whereas the assays were done independently of Fig 3. The error bars show the standard error of the mean (SEM) of five independent experiments. (PDF) [file pgen.1008103.s003.pdf]

Fig.S4 Horigome and Unozawa et al, 2019

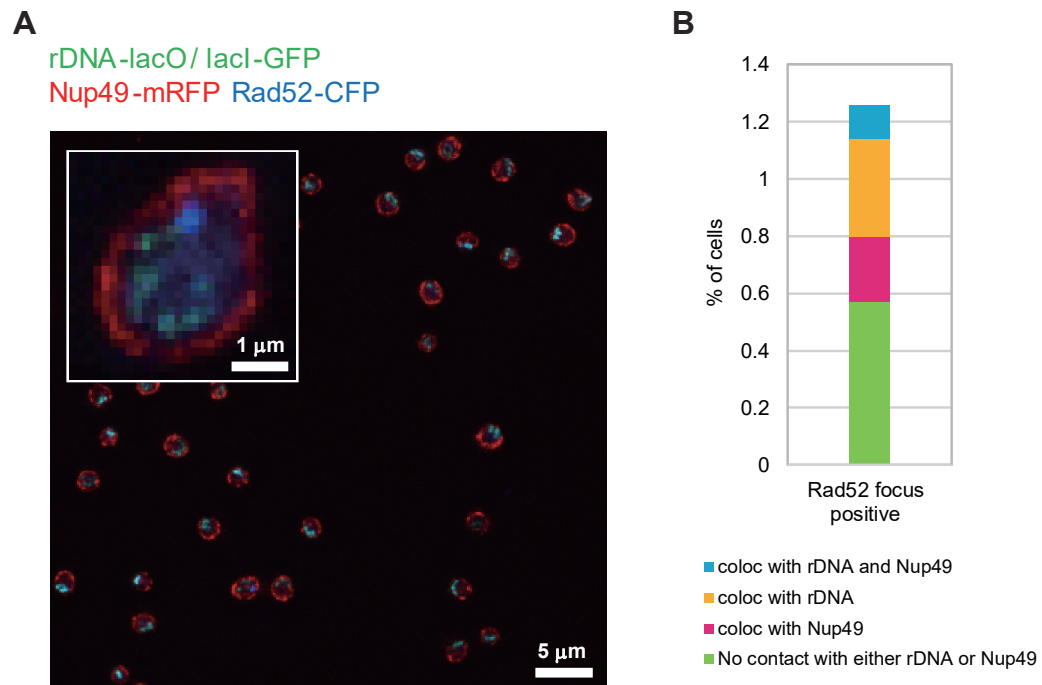

Supplement: S4 Fig — (A) Colocalization of a Rad52-CFP, Nup49-mRFP and LacI-GFP / rDNA-lacO. A representative image is shown. A magnified window shows the colocalization of Rad52-CFP and rDNA. (B) Through-focus stack images of 12 0.3 μm steps were used to determine the colocalization. Rad52-CFP position was compared with LacI-GFP/ rDNA-lacO and Nup49-mRFP. We defined the following three situations as colocalization: fully overlapping, partially overlapping, and juxtaposition. (PDF) [file pgen.1008103.s004.pdf]
